# Combined Inter- and Intrafractional Plan Adaptation Using Fraction Partitioning in Magnetic Resonance-guided Radiotherapy Delivery

**DOI:** 10.7759/cureus.2434

**Published:** 2018-04-05

**Authors:** Frank Lagerwaard, Omar Bohoudi, Shyama Tetar, Marjan A Admiraal, Tezontl S Rosario, Anna Bruynzeel

**Affiliations:** 1 Radiation Oncology, VU University Medical Center, Amsterdam, The Netherlands

**Keywords:** mrgrt, adaptive, interfraction, intrafraction, pancreatic cancer, smart, partitioning, re-optimization

## Abstract

Magnetic resonance-guided radiation therapy (MRgRT) not only allows for superior soft-tissue setup and online MR-guidance during delivery but also for inter-fractional plan re-optimization or adaptation. This plan adaptation involves repeat MR imaging, organs at risk (OARs) re-contouring, plan prediction (i.e., recalculating the baseline plan on the anatomy of that moment), plan re-optimization, and plan quality assurance. In contrast, intrafractional plan adaptation cannot be simply performed by pausing delivery at any given moment, adjusting contours, and re-optimization because of the complex and composite nature of deformable dose accumulation. To overcome this limitation, we applied a practical workaround by partitioning treatment fractions, each with half the original fraction dose. In between successive deliveries, the patient remained in the treatment position and all steps of the initial plan adaptation were repeated. Thus, this second re-optimization served as an intrafractional plan adaptation at 50% of the total delivery. The practical feasibility of this partitioning approach was evaluated in a patient treated with MRgRT for locally advanced pancreatic cancer (LAPC).

MRgRT was delivered in 40Gy in 10 fractions, with two fractions scheduled successively on each treatment day. The contoured gross tumor volume (GTV) was expanded by 3 mm, excluding parts of the OARs within this expansion to derive the planning target volume for daily re-optimization (PTV_OPT_). The baseline GTVV_95%_ achieved in this patient was 80.0% to adhere to the high-dose constraints for the duodenum, stomach, and bowel (V_33 Gy_ <1 cc and V_36 Gy_ <0.1 cc). Treatment was performed on the MRIdian (ViewRay Inc, Mountain View, USA) using video-assisted breath-hold in shallow inspiration. The dual plan adaptation resulted, for each partitioned fraction, in the generation of Plan_PREDICTED1_, Plan_RE-OPTIMIZED1 _(inter-fractional adaptation), Plan_PREDICTED2_, and Plan_RE-OPTIMIZED2_ (intrafractional adaptation). An offline analysis was performed to evaluate the benefit of inter-fractional versus intrafractional plan adaptation with respect to GTV coverage and high-dose OARs sparing for all five partitioned fractions.

Interfractional changes in adjacent OARs were substantially larger than intrafractional changes. Mean GTV V_95%_ was 76.8 ± 1.8% (Plan_PREDICTED1_), 83.4 ± 5.7% (Plan_RE-OPTIMIZED1_), 82.5 ± 4.3% (Plan_PREDICTED2_),and 84.4 ± 4.4% (Plan_RE-OPTIMIZED2_). Both plan re-optimizations appeared important for correcting the inappropriately high duodenal V_33 Gy_ values of 3.6 cc (Plan_PREDICTED1_) and 3.9 cc (Plan_PREDICTED2_) to 0.2 cc for both re-optimizations. To a smaller extent, this improvement was also observed for V_25 Gy_ values. For the stomach, bowel, and all other OARs, high and intermediate doses were well below preset constraints, even without re-optimization. The mean delivery time of each daily treatment was 90 minutes.

This study presents the clinical application of combined inter-fractional and intrafractional plan adaptation during MRgRT for LAPC using fraction partitioning with successive re-optimization. Whereas, in this study, interfractional plan adaptation appeared to benefit both GTV coverage and OARs sparing, intrafractional adaptation was particularly useful for high-dose OARs sparing. Although all necessary steps lead to a prolonged treatment duration, this may be applied in selected cases where high doses to adjacent OARs are regarded as critical.

## Introduction

Magnetic resonance-guided radiation therapy (MRgRT) has become a clinical reality with a number of centers reporting feasibility and preliminary clinical results [1-3]. In addition to superior soft-tissue setup and online MR-guidance during delivery, an attractive option with MRgRT could be to perform a daily plan re-optimization, or adaptation, prior to the delivery of each fraction. At our center, respiratory-gated MRgRT is delivered during subsequent breath-hold spells in combination with real-time MR guidance of the gross tumor volume (GTV). This approach allows for ensuring adequate target coverage, even with the use of minimal GTV to planning target volume (PTV) margins. Interfractional plan adaptation is routinely performed for each patient and each fraction at our center. Several recent publications and presentations have highlighted the relevance of interfractional plan adaptation, for instance, for prostate, adrenal, and pancreatic tumors [1-2,4-5]. In contrast, however, the extent of intrafractional changes in the position and volume of surrounding organs at risk (OARs) during radiation delivery, and thereby the relevance of intrafractional plan adaptation, is largely unknown. At our center, MRgRT is delivered in the form of intensity modulated radiotherapy (IMRT) using the MRIdian system (ViewRay Inc, Mountain View, USA), resulting in highly conformal treatment plans. Using the current software, however, intrafractional plan adaptation cannot be simply performed by pausing delivery at any given moment, adjusting contours, and re-optimization because of the complex and composite nature of deformable dose accumulation.

To overcome this limitation, we developed and investigated a practical workaround by partitioning treatment fractions at a fixed interval, each with half of the original fraction dose. In between successive deliveries, repeat MR imaging (MRI), OAR re-contouring, and plan re-optimization were performed with the patient remaining in the treatment position. Thus, this second re-optimization serves as an intrafractional plan adaptation at 50% of the total radiation delivery. The practical feasibility of this partitioning approach was evaluated in a patient treated with stereotactic MR-guided radiation therapy (SMART) for locally advanced pancreatic cancer (LAPC).

## Case presentation

**Figure 1:**
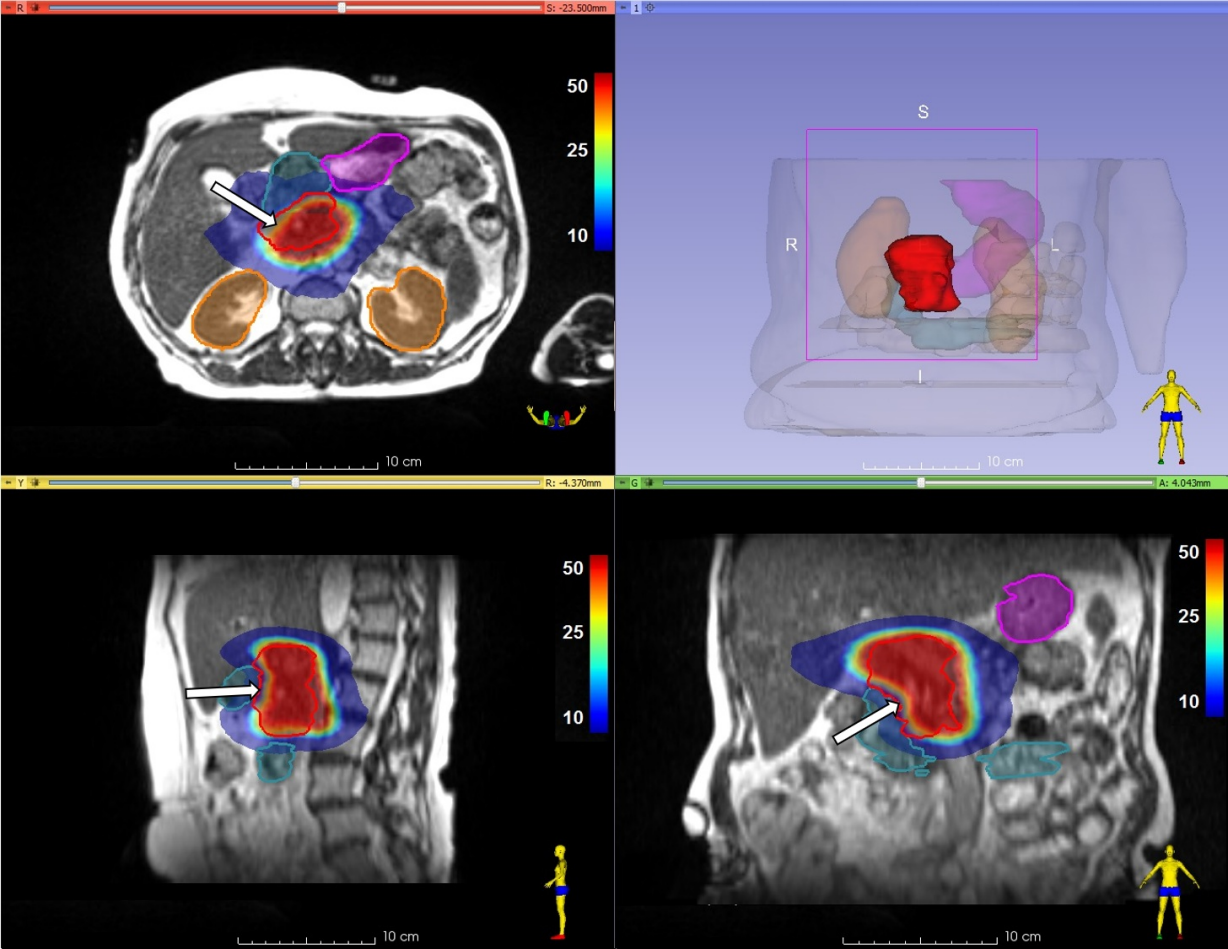
Baseline IMRT plan Baseline IMRT plan with dose (Gy) in color wash. Relative PTV_OPT _underdosing can be seen at the border between the PTV_OPT _and the duodenum (arrows), in order to adhere to high-dose OARs constraints. IMRT=  intensity-modulated radiotherapy, PTV_OPT_= planning treatment volume for re-optimization, OARs= organs at risk PTV_OPT_ = red contour, Duodenum = cyan color wash, Stomach = purple color wash, Kidneys = orange color wash

The patient is a 66-year-old female, who was diagnosed with LAPC in March 2017 and was treated with Folfirinox. Chemotherapy was discontinued after three courses as a result of severe toxicity, at which time, diagnostic computed tomography (CT) scans showed a stable disease. She was referred by her medical oncologist for stereotactic radiotherapy in the form of MRgRT. After performing a simulation CT and MR scan on MRIdian, both in shallow inspiration breath-hold, contouring of the GTV and relevant OARs was performed in collaboration with a radiologist specialized in gastrointestinal radiology, No separate margins for the clinical target volume (CTV) were applied (GTV=CTV), and the PTV for daily re-optimization (PTV_OPT_) was defined by adding an isotropic 3 mm margin to the GTV, excluding parts of OARs within this expansion. The standard fractionation scheme for MRgRT in LAPC at our center is 40Gy in five fractions, in three fractions per week. In this case, the 40Gy was prescribed in 10 fractions, with two fractions scheduled immediately successive on each treatment day. The generation of a robust baseline treatment plan (BL) (Figure 1), also for use in daily adaptation, was performed as previously described [1].

The patient was positioned with one arm up using an MR-compatible positioning board. A new high-resolution MR scan in shallow inspiration was acquired and aligned with the simulation GTV. After deformable contour propagation of the OARs from the BL plan, the OARs contours were manually adjusted in the first 3 cm around the PTV_OPT_. Subsequently, the BL plan was recalculated on the anatomy of the moment (Plan_PREDICTED1_) and re-optimized using the same number and direction of beams (Plan_RE-OPTIMIZED1_; plan A). This approach of maintaining the original beam setup increases the speed of plan adaptation, facilitates patient-specific QA, and can be performed within minutes, with the patient remaining in treatment position. After patient-specific plan quality assurance (QA), radiation delivery (4Gy) was performed under patient-controlled breath-hold conditions with video feedback.

Immediately after the completion of plan A, the high-resolution MR imaging in breath-hold was repeated, with the patient remaining in the treatment position. After re-alignment on the GTV, because of a different breath-hold, deformed OARs were again manually adjusted, if needed. This time, however, instead of the BL plan, plan A was used as a primary imaging set. This allows for faster recontouring because only intrafractional OARs changes needed to be adjusted. Calculation of plan A on the repeated MR scan (Plan_PREDICTED2_) was again followed by plan re-optimization (Plan_RE-OPTIMIZED2_; plan B) and QA, which was subsequently delivered (4Gy) using the same breath-hold conditions. The average total duration of delivering such a partitioned, twice re-optimized treatment fraction was approximately 90 minutes in comparison to 75 minutes for our standard single re-optimized treatment.

**Figure 2:**
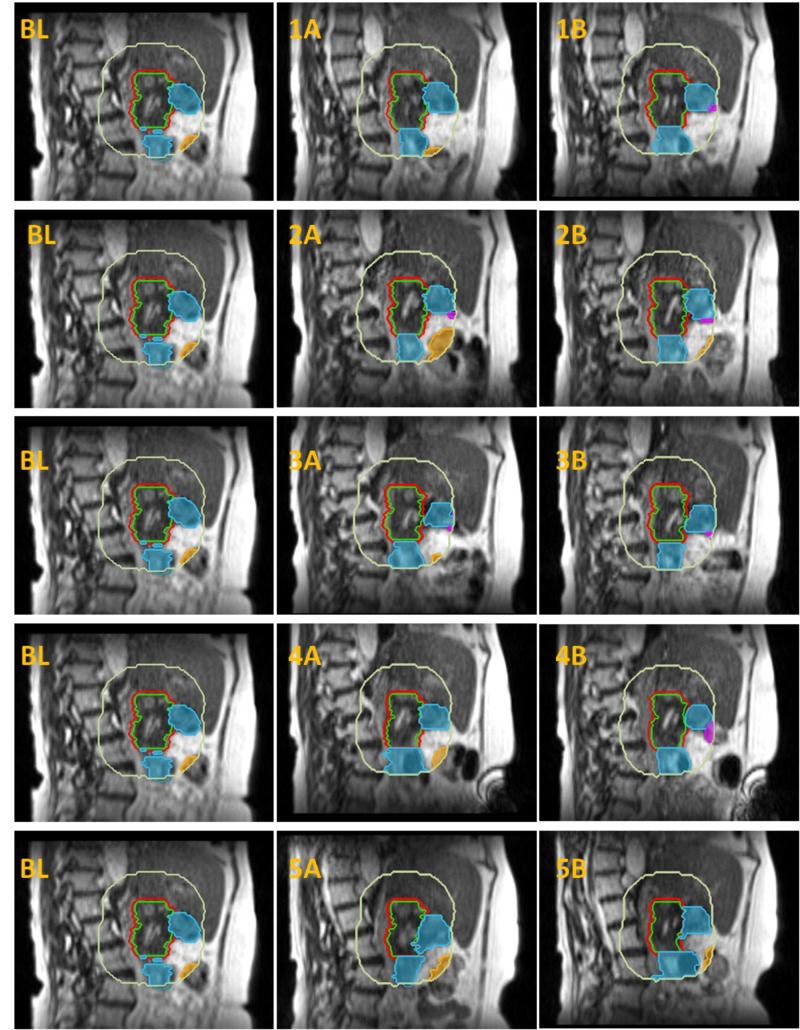
Anatomical changes Anatomical changes in the position of relevant OARs in the first 3 cm outside the PTV_OPT_, shown in a sagittal plane through the center of the GTV. The simulation MR is shown in the left panels. The middle panels show the anatomy prior to the delivery of plan A (fractions 1A-5A, respectively). The right panels illustrate the OARs position after the delivery of 4Gy; prior to the delivery of plan B (fractions 1B-5B, respectively). OARs = organs at risk, PTV_OPT _= planning target volume for re-optimization, GTV = gross tumor volume GTV = green contour, PTV_OPT _= red contour, Duodenum = cyan color wash, Stomach = purple color wash, Bowel = orange color wash, 3 cm Ring = light yellow contour

The extent of changes in the OARs surrounding the PTV between the simulation scan and the pretreatment scan (interfractional) was substantially larger than in between both partitioned fractions, shown for the sagittal planes in Figure 2 (corresponding axial and coronal planes in Figures 6-7, Appendix).

**Figure 3:**
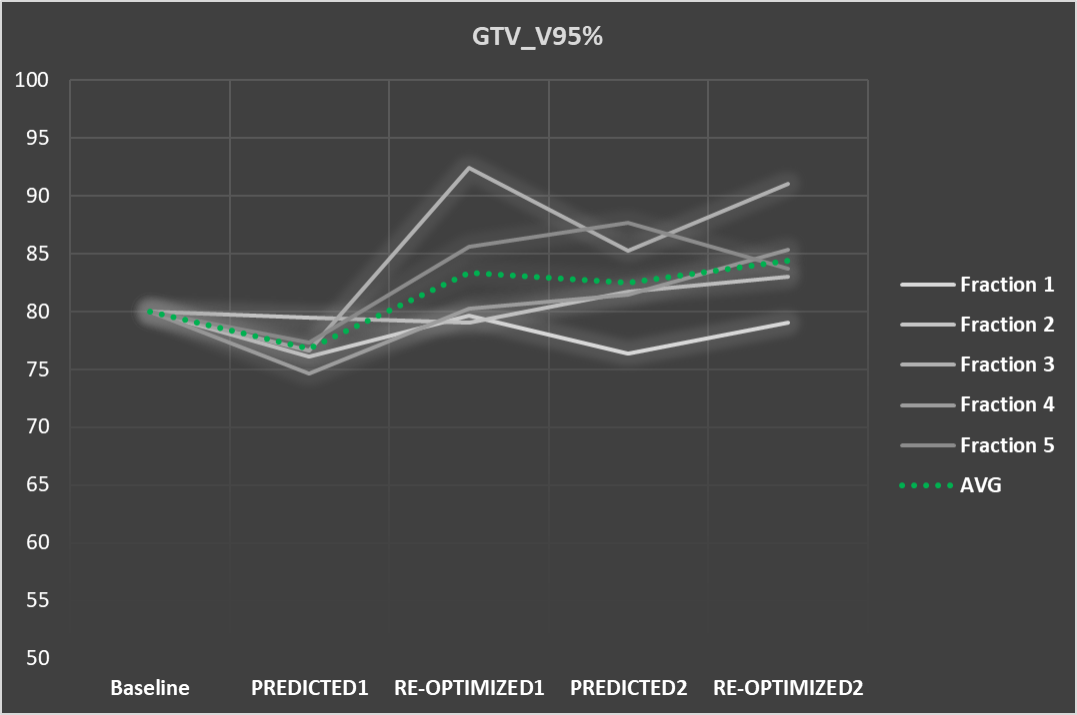
GTV coverage (V95%) GTV coverage (V95%) for the five fractions. Target coverage in PLAN_RE-OPTIMIZED1_ is clearly improved in comparison to PLAN_PREDICTED1_. After repeat setup on the GTV, the second plan adaptation had a limited effect on target coverage. Average coverage is seen as a green dotted line. GTV = gross tumor volume

An offline analysis was performed to evaluate the benefit of interfractional (Plan_PREDICTED1_ vs. Plan_RE-OPTIMIZED1_) versus intrafractional plan adaptation (Plan_PREDICTED2_ vs. Plan_RE-OPTIMIZED2_) with respect to target coverage and high-dose OARs sparing for all five partitioned fractions. In comparison to the baseline GTV V95% of 80.0%, the average GTV V95% in the partitioned plans was 76.8 ± 1.8% (Plan_PREDICTED1_), 83.4 ± 5.7% (Plan_RE-OPTIMIZED1_), 82.5 ± 4.3% (Plan_PREDICTED2_), and 84.4 ± 4.4% (Plan_RE-OPTIMIZED2_) (Figure 3).

**Figure 4:**
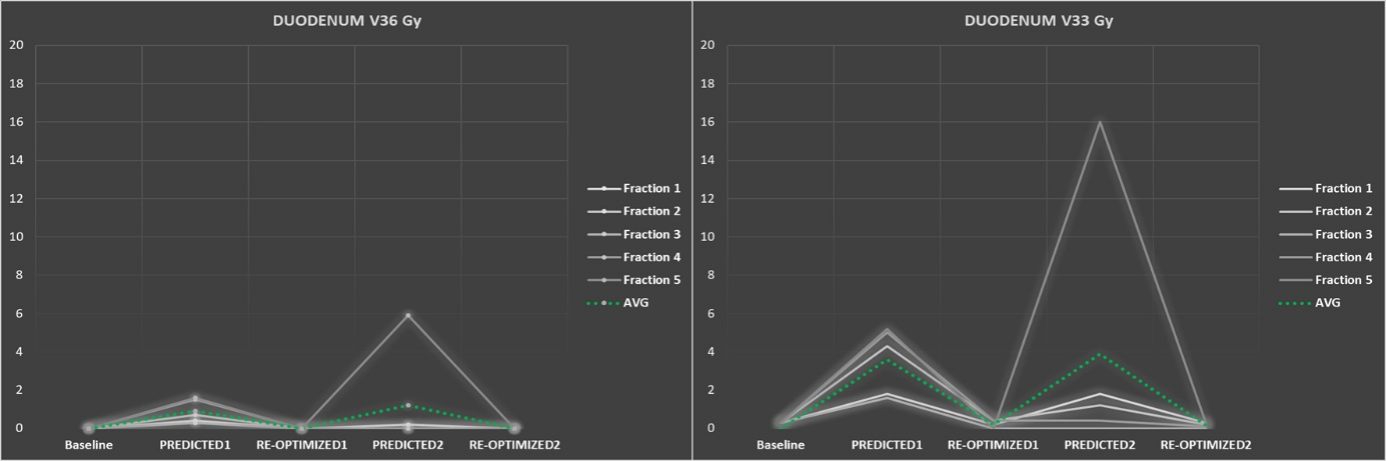
Duodenal high dose Results of inter- and intrafractional plan adaptation for high doses to the duodenum. Both re-optimization steps clearly correct any high duodenal V_36 Gy_ and V_33 Gy_ for all fractions. Green dotted line indicates average for all fractions.

Both plan re-optimizations appeared important for substantially restricting the duodenal high doses (V_36 Gy_/V_33 Gy_). For each fraction, duodenal V_36 Gy_ was <0.1 cc after the first and second re-optimizations (Figure 4, left panel). As per institutional protocol, the duodenal V_33 Gy_ should be ≤1 cc. Inappropriately high mean V_33 Gy_ values of 3.6 cc (Plan_PREDICTED1_) and 3.9 cc (Plan_PREDICTED2_) were corrected to a mean of 0.2 cc for both re-optimizations (Figure 4, right panel). To a lesser extent, this improvement was also observed for V_25 Gy_ values (data not shown). For the stomach and bowel, as well as other OARs at a bigger distance (kidneys, liver, and spinal cord), all high and intermediate doses were well below preset constraints, even without re-optimization.

## Discussion

With the implementation of MRgRT, real-time plan adaptation has become a clinical reality, which has been reported to increase target coverage and/or OARs sparing for various indications. The extent of intrafractional changes in relevant OARs during radiation delivery and, consequently, the need for intrafractional plan adaptation, is currently unknown. This case report describes a first attempt to quantify the relative importance of inter-fractional and intrafractional plan adaptation. Because our current software version does not allow for intrafractional plan adaptation at any given moment due to the absence of dose accumulation, a workaround using fixed fraction partitioning is needed to perform intrafractional plan adaptation, in this case, at 50% of total fraction delivery. In this simplified manner, dose accumulation is feasible by prescribing an adequate GTV coverage and adhering to high-dose OARs constraints for each partitioned fraction.

Our case underscores the importance of inter-fractional plan adaptation, visualized by substantial changes in OARs between the simulation scan and the pre-fractional MR scans, as well as by the increase in GTV coverage and the decrease in high doses to OARs after the first plan re-optimization. This observed relevance of inter-fractional plan adaptation may be greater because of the use of small (3 mm) GTV to PTV margins, steep dose gradients, generating a new PTV_OPT_ for each fraction, and, certainly, the relatively lengthy delivery procedure.

**Figure 5:**
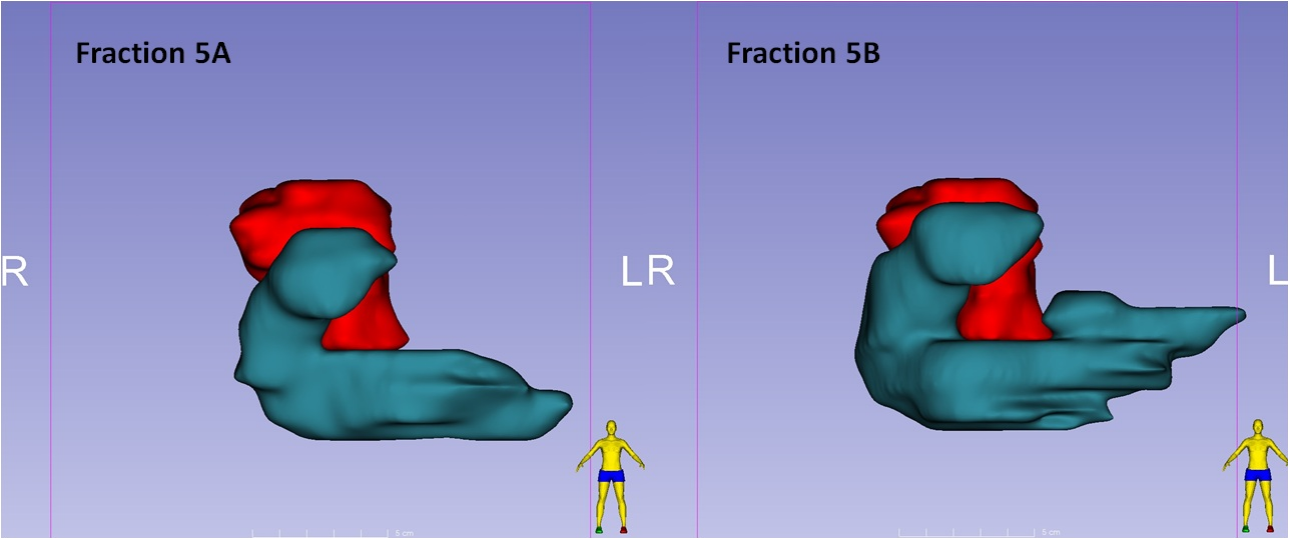
Intrafractional changes duodenum-PTV 3D image of the PTV_OPT _(red volume) and duodenum (cyan volume) for fractions A and B of fraction 5, showing a significant change in the anatomy. This intrafractional changes resulted in a high dose to the duodenum in the PLAN_PREDICTED2_, which was subsequently corrected by the second re-optimization. PTV_OPT _= planning target volume for re-optimization

**Figure 6:**
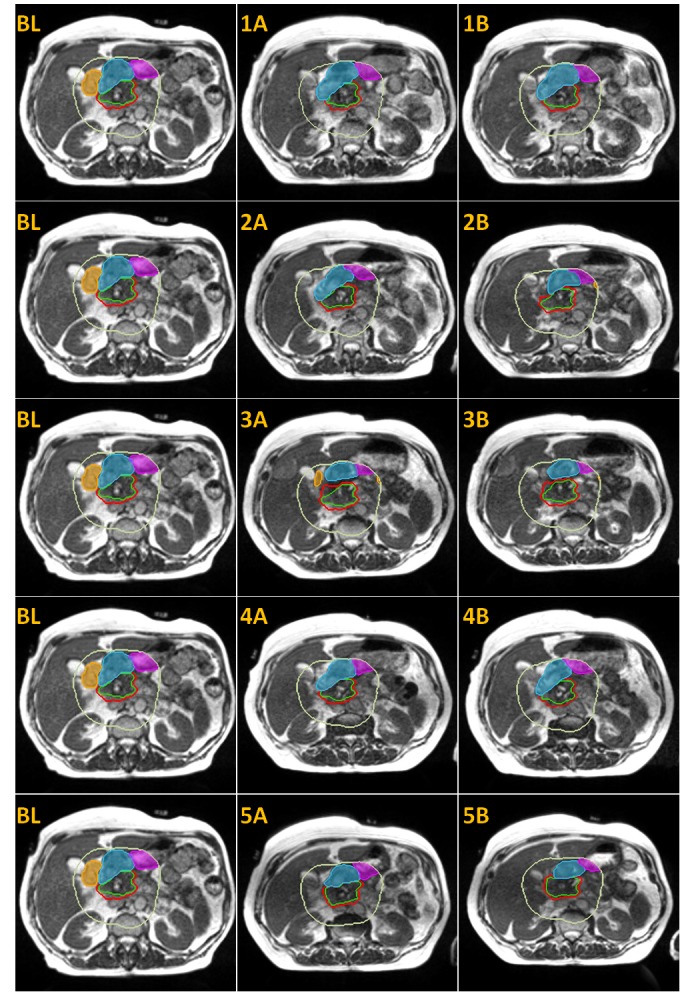
Anatomical changes (axial) Anatomical changes in the positions of relevant OARs in the first 3 cm outside the PTV_OPT_, shown in an axial plane through the center of the GTV. The simulation MR is shown in the left panels. The middle panels show the anatomy prior to the delivery of plan A (fractions 1A-5A, respectively). The right panels illustrate the OARs position after the delivery of 4Gy; prior to the delivery of plan B (fractions 1B-5B, respectively). OARs = organs at risk, PTV_OPT _= planning target volume for re-optimization, GTV = gross tumor volume GTV = green contour, PTV_OPT _= red contour, Duodenum = cyan color wash, Stomach = purple color wash, Bowel = orange color wash, 3 cm ring = light yellow contour

**Figure 7:**
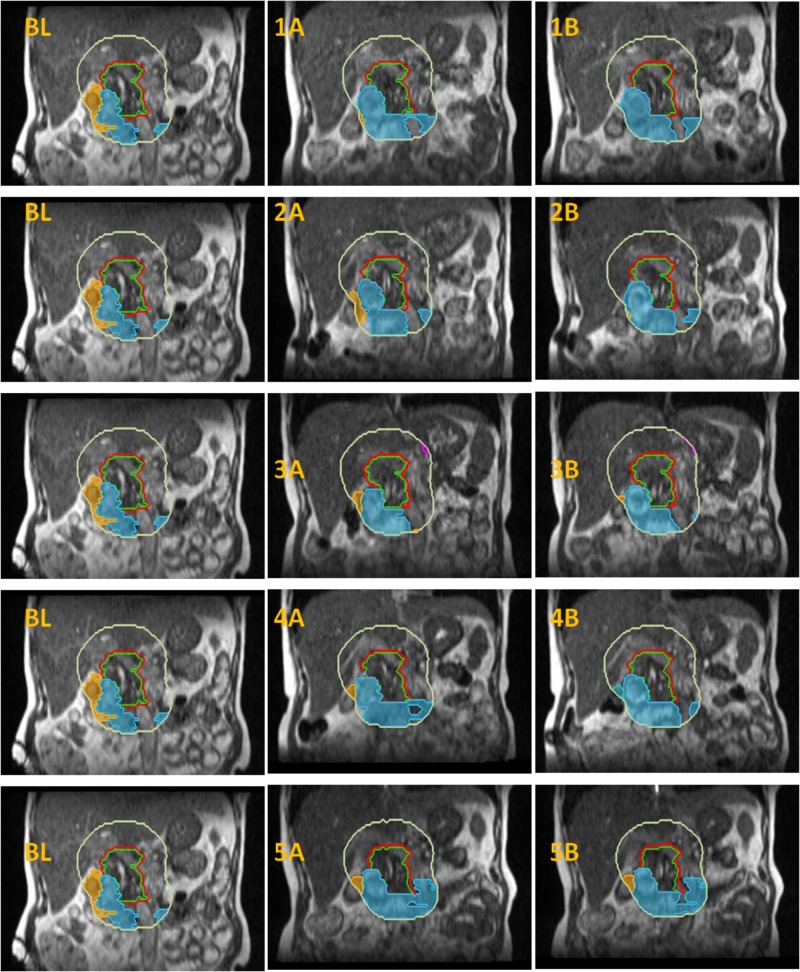
Anatomical changes (sagittal) Anatomical changes in the position of relevant OARs in the first 3 cm outside the PTV_OPT_, shown in a coronal plane through the center of the GTV. The simulation MR is shown in the left panels. The middle panels show the anatomy before the delivery of plan A (fractions 1A-5A, respectively). The right panels illustrate the OARs position after the delivery of 4Gy; prior to the delivery of plan B (fractions 1B-5B, respectively). OARs = organs at risk, PTV_OPT _=planning target volume for re-optimization, GTV = gross tumor volume GTV = green contour, PTV_OPT _= red contour, Duodenum = cyan color wash, Stomach = purple color wash, Bowel = orange color wash, 3 cm ring = light yellow contour

Our preliminary results regarding intrafractional plan adaptation are less clear-cut. Intrafractional plan adaptation had only a modest effect on target coverage, however, it did decrease high-doses to the duodenum in several fractions. This could be expected after repeat setup on the GTV with the patient remaining in the treatment position. Furthermore, the first re-optimized plan was taken as a reference for the second re-optimization, which reflects the anatomy of that day better than the BL plan. A single fraction showed an extreme benefit of the second re-optimization, which was due to expansion and displacement of the duodenum during the delivery of plan A. A 3D image of the PTV_OPT_ and the duodenum illustrates this intrafractional change better than the single slice coronal view (Figure 5). Reassuringly, such anatomical changes did not occur systematically, and the cumulative dosimetric consequences will be limited. This finding does, however, illustrate the potential danger of re-optimizing followed by re-normalizing to the limit of critical OARs constraints since such intrafractional changes may occur. Some limitations of our analysis have to be mentioned here. Because simulation, as well as all partitioned fractions, have been performed during patient-controlled shallow-inspiration breath-hold, small differences in respiratory phase may exist both in the analysis of inter-fractional and intrafractional plan adaptation. In addition, small contouring variations may influence particularly the high-dose OAR results for all parts of this analysis. We have tried to minimize the latter by having the same specialized radiation oncologist performing the recontouring for all fractions. Finally, the results may be different for other approaches of re-optimization, for example, in cases where a new plan with different beam numbers and directions is generated for each adaptation.

## Conclusions

To the best of our knowledge, this case presentation is the first clinical application of combined inter-fraction and intrafraction plan adaptation during MRgRT. In order to achieve this, we have used fraction partitioning with successive re-optimization. Whereas inter-fractional plan adaptation appears to benefit both GTV coverage and OARs sparing, intrafraction plan adaptation was found to be particularly useful for OARs sparing in this specific case we described. Although all necessary steps result in a prolonged treatment duration, this may be used in selected cases where the high doses to adjacent OARs are regarded to be critical. Intrafractional plan adaptation will benefit from future three-dimensional (3D) real-time MR imaging, as well as from software improvements to allow faster re-optimization and dose-accumulation.
